# Impacted Ectopic Maxillary Incisor at the Margins of an Oronasal Palatal Fistula

**DOI:** 10.7759/cureus.55303

**Published:** 2024-03-01

**Authors:** Varun H Kashyap, Veena Kumari, Niraj Bhalara

**Affiliations:** 1 Plastic and Reconstructive Surgery, All India Institute of Medical Sciences, Patna, Patna, IND; 2 Plastic surgery, All India Institute of Medical Sciences, Patna, Patna, IND; 3 Plastic Surgery, All India Institute of Medical Sciences, Patna, Patna, IND

**Keywords:** two-flap repair, cleft lip and palate, cleft palate fistula, bardach, fistula management, palatoplasty

## Abstract

An oronasal fistula is one of the most common complications that can occur after cleft palate surgeries. Some of the reasons for the failure of repair are the closure of palatal flaps under tension, vascular compromise, and infection. We present a case of a 10-year-old patient who experienced nasal regurgitation during feeding, four years after undergoing a redo palatoplasty. The reason was identified as an impacted maxillary incisor located at the fistula site. The patient was managed with the closure of the oronasal palatal fistula, with a two-layered repair technique using bilateral mucoperiosteal flaps after the removal of the impacted tooth.

## Introduction

Fistulas are more frequently observed in patients with severe clefts, according to research [1]. In a study of 211 patients with cleft palates, a fistula rate of 12.8% was discovered [2]. Another study found that palatal repair before 12 months of age was linked to a lower incidence of fistula formation, compared to repairs conducted between 12 and 25 months of age [3]. Fistulas can be classified into small (< 2 mm), medium (3-5 mm), or large (> 5 mm) based on their size [4]. External causes of these fistulas include tension, absent layered repair, and poor surgical technique.

## Case presentation

A 10-year-old child accompanied by his mother visited the Plastic Surgery outpatient department with complaints of nasal regurgitation for three years and an impacted foreign body in the palate for two years. The mother provided a history of the complete cleft of the lip, palate, and alveolus on the left side. Lip repair was performed at the age of four months, and primary palatoplasty was done at the age of one year. Later, the mother noticed a gap in the palate three months after surgery, for which a redo palatoplasty was done eight months after the first surgery. The operative notes of redo palatoplasty mentioned the two-flap palatoplasty technique for the same.

**Figure 1:**
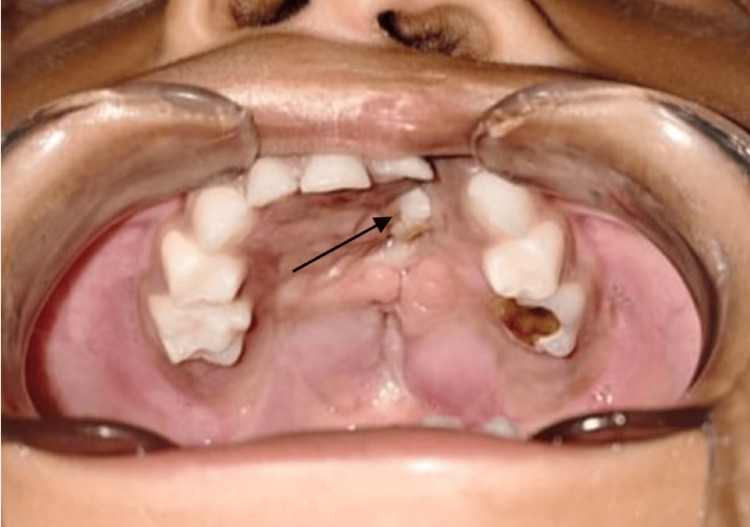
Repair for congenital complete left-sided cleft lip and palate (Veau III according to the Veau classification for cleft palate severity). The arrow shows the impacted hard swelling in the region of the fistula.

Upon examination of the palate, a single oro-nasal fistula measuring 12 mm in length and 18 mm in width was located on the left side of the anterior palate. Additionally, a white hard swelling of 4 mm x 2 mm in size with an irregular surface was felt at the posterior edge of the fistula, which appeared to be a tooth (Figure 1).

## Discussion

Management

**Figure 2:**
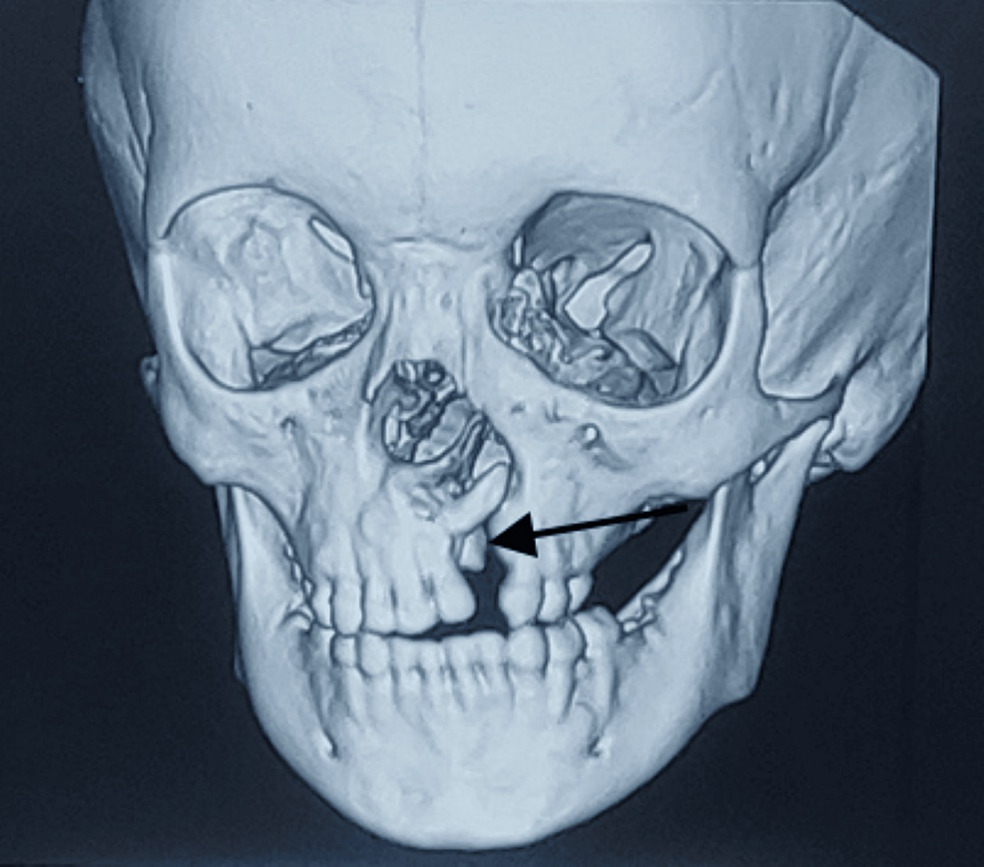
Non-contrast CT face with 3D reconstruction showing tooth impacted within the cleft at the alveolar process of the maxilla. The arrow shows the impacted maxillary incisor tooth.

A plain CT scan of the face was carried out, which included a 3D reconstruction. The results showed a defect on the left side of the hard palate in the maxillary alveolus, measuring 3.1 x 0.9 centimeters, with the left lateral incisor facing superolaterally into the left nasal cavity through the defect (Figure 2).

Oronasal fistulas after palatoplasty occur most commonly at the junction of the hard and soft palates [5]; in our case, a fistula over the hard palate was observed. 

The hard palate fistulas should be closed in two layers preferably. However, three layered closures have been reported, with the intermediate layer being bone, cartilage, or acellular dermal matrix [6]. A complete redo palatoplasty is required in most of the cases [7].

Surgical correction

**Figure 3:**
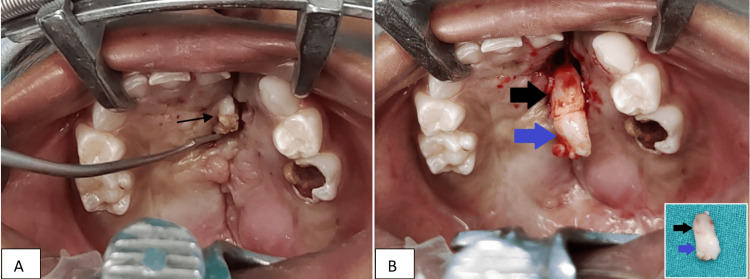
A) Intraoperative picture shows the oronasal fistula with impacted left maxillary lateral incisor. B) The big blue arrow shows the crown of the incisor, and the big black arrow shows the root of the incisor. The picture in the lower right corner shows the completely extracted tooth.

Under general anesthesia, the margins of the fistula and the hard palate were infiltrated with a 1:1,00,000 adrenaline solution. The impacted maxillary lateral incisor was extracted, and it was confirmed anatomically to be a tooth as it contained cementum and pulp in the impacted section (Figure 3B). The socket was cauterized to prevent any further eruption of permanent teeth.

**Figure 4:**
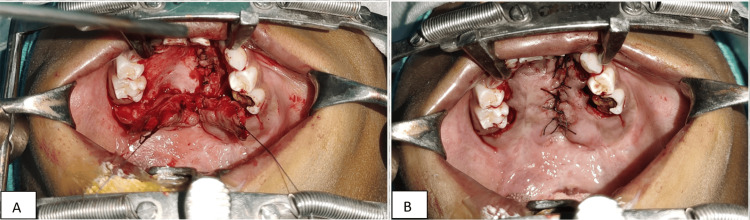
A tension-free closure of the nasal layer and the oral layer. A) Elevated bilateral mucoperiosteal flaps. B) Closure of the oral layer.

Fistula repair was done in two layers with nasal mucosal repair and oral mucosa closure with posteriorly based mucoperiosteal flaps from both sides (Figure 4).

Postoperative management

The patient’s mother was advised on a diet plan that included a clear liquid diet for four weeks, which reduces the physical stress on the palate, water after every feed, good oral hygiene, and diluted chlorhexidine mouthwash [8].

IV antibiotics and analgesics were given for five days, and the patient was discharged on postoperative day eight after ensuring an absence of any local or general complications. A soft diet was allowed after four weeks. A follow-up revealed no symptoms of nasal regurgitation nor any suture line complications. A normal oral diet was advised after eight weeks of the postoperative period.

**Figure 5:**
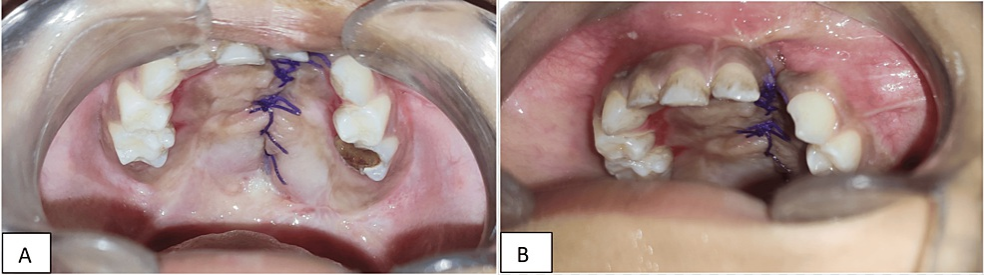
A healthy stitch line and oral mucosa. A) Suture line at two weeks of repair. B) Suture line at four weeks of repair.

Figure 5 shows a healthy stitch line and oral mucosa postoperatively.

## Conclusions

The management of palatal fistulas following primary palatoplasty complications is a challenging surgical procedure due to mucosal scarring, limited virgin mucosa, and a high recurrence rate. No documented literature identifies an impacted maxillary lateral incisor, or any ectopically erupting tooth as a contributing factor to palatal fistulas. During primary palatoplasty, the displacement of alveolar mucosa carrying the tooth bud over the cleft margin might be a possibility that led to its impaction at the palate site. As the permanent maxillary lateral incisor erupts at eight to nine years of age, the patient’s ectopic eruption of the incisor might have led to a palatal fistula.

To ensure successful repair, it is crucial to correctly orient the gingival and palatal mucoperiosteal areas, maintain functional separation between the oral and nasal cavities through a layered repair, achieve tensionless closure, and preserve the vascularity of the raised flaps.
